# Transcriptome and Metabolome Analyses of Flavonoid Biosynthesis During Berry Development of Muscadine Grape (*Vitis rotundifolia* Michx)

**DOI:** 10.3390/plants14132025

**Published:** 2025-07-02

**Authors:** Qiaofeng Yang, Changlin Li, Yan Wang, Xian Pei, Aixin Wang, Li Jin, Linchuan Fang

**Affiliations:** Institute of Forestry and Fruit Tree, Wuhan Academy of Agricultural Science, Wuhan 430065, China; qfyang2022@126.com (Q.Y.); lichanglin79@sina.com (C.L.); sophie_2003@126.com (Y.W.); zaiyunren@126.com (X.P.); aixinwang@126.com (A.W.); jinli@126.com (L.J.)

**Keywords:** muscadine grape, metabolome, flavonoids biosynthesis, anthocyanidins, proanthocyanins

## Abstract

Flavonoids play a crucial role in plant development, resistance, and the pigmentation of fruits and flowers. This study aimed to uncover the mechanism of flavonoid biosynthesis and fruit coloring in muscadine grapes. Two muscadine genotypes (Paulk and Supreme) were investigated via metabolomic and transcriptomic analysis during three developmental stages (bunch closure, veraison stage, and ripening stage). A total of 314 flavonoids were identified, with flavones and flavonols being the primary constituents. The contents of many differentially accumulated metabolites (DAMs) were higher at the veraison stage. The total anthocyanin content was upregulated during berry development, with the dominant type of anthocyanidin-3,5-O-diglucoside. Proanthocyanins accumulated higher levels in the ripening stage of Paulk than Supreme. Transcriptomic analyses revealed that over 46% of the DEGs exhibited higher expression levels in the bunch closure stage. Moreover, *phenylalanine ammonia-lyase* (*PAL*), *cinnamyl 4-hydroxylase* (*C4H*), and *coumaryl CoA ligase* (*4CL*) genes were upregulated during berry development, suggesting they promote second metabolites biosynthesis. The upregulation of *dihydroflavonol 4-reductase* (*DFR*) and *leucoanthocyanin reductase* (*LAR*) may related to the higher levels of PA in Paulk. *Anthocyanidin synthase* (*ANS*) and *UDP-glucose:flavonoid-3-O-glucosyltransferase* (UFGT) showed higher expression levels in the ripening stage, which may relate to the accumulation of anthocyanidins. This study provides comprehensive insights into flavonoid metabolism and berry coloration in *Vitis rotundifolia*.

## 1. Introduction

Grapes (*Vitis* L.) are among the earliest domesticated and most economically significant fruit crops worldwide [1]. The *Vitis* genus is divided into two subgenera: *Euvitis* Planch. and *Muscadinia* Planch. The majority of grapes cultivated for fruit production are either *Vitis vinifera* L. species or hybrids of *V. vinifera* and *V. labrusca* L., which belong to the *Euvitis* subgenus [2]. The muscadine grape (*Muscadinia rotundifolia* Michx. or *V. rotundifolia* Michx.) belongs to the *Muscadinia* subgenus [3,4]. The basic chromosome number of the muscadine grape (x = 20) exceeds the 19 basic chromosomes of *V. vinifera* [2]. Muscadine grapes have been cultivated in the southeastern United States for over 400 years [5]. They have excellent environmental adaptability, particularly in hot and humid climates, and high resistance to major grape fungal diseases [6]. Numerous varieties of muscadine grapes have been introduced to China, including Guangxi, Yunnan, and Fujian provinces [7]. Furthermore, these grapes are rich in plant secondary metabolites such as resveratrol [8]. Muscadine grapes are the only genus containing ellagic acid, which possesses numerous health benefits, including antioxidant, anticancer, antibacterial, and anti-inflammatory properties [9,10].

Muscadine grapes (*Vitis rotundifolia*) are known to be potential sources of antioxidants due to high concentrations of bioactive phytochemicals, such as flavonoids and other polyphenol compounds [11,12]. Flavonoids, a class of secondary metabolites prevalent in plants, play crucial roles in various biological processes and plant–environmental interactions [13]. Flavonoids are key compounds for berry and wine quality in grapes [14]. These C6-C3-C6 compounds are divided into six classes based on the oxidation state of the C ring: flavanones, flavones, flavonols, isoflavones, flavan-3-ols, and anthocyanins [13,15]. Modification reactions, such as glycosylation, acylation, and methylation, further enhance the diversity of flavonoids. Flavonoid C-glycoside has been reported to be the main modification type accumulated in grapes [16]. The composition and content of flavonoids during fruit development are different. It has been reported that flavonoids accumulated peaks at the rapid growth phase [17].

In plants, flavonoids contribute to a range of flower colors from yellow to purple [18]. Anthocyanins are the primary components of red to purple pigments, and flavonols are not only the main components of colorless to light-yellow pigments but also increase color by stabilizing the colored form of the anthocyanin molecule through co-pigmentation [19,20]. Six main anthocyanins have been identified based on different substituents: cyanidin, delphinidin, peonidin, petunidin, pelargonidin, and malvidin [13]. These six anthocyanidins contribute different colors, with pelargonidin being bright red, cyanidin red, and delphinidin purple or blue [18,19]. Anthocyanidins are unstable and require modification into anthocyanins by glycosylation and acylation. These anthocyanins were then transported and deposited in vacuoles [19]. The total anthocyanin content, responsible for skin color, increases during grape maturation [21]. Malvidin derivatives are the primary anthocyanins in grapes (*Vitis vinifera*) [16]. Flavonols accumulate exclusively in the berry skin of ripening grapes but are not in Chardonnay and Shiraz’s developing seeds or flesh (*Vitis vinifera*) [20]. Proanthocyanidins (PAs), ubiquitous groups of plant phenolics, are oligomers or polymers of flavan-3-ols units. PAs extracted from grapes have been widely used as nutritional supplements, and synthesis occurs from anthesis until the onset of ripening [22,23].

The flavonoid biosynthesis pathway has been well-characterized, and MYB transcription factors regulate it. In purple grapes, *VlMYBA1*-*3*, *VlMYBA1*-*2*, and *VlMYBA2* have been reported to regulate anthocyanin synthesis [24]. *VvMYBA1* and *VvMYBA2* promote anthocyanin synthesis, and the simultaneous mutation of these two isoforms results in the complete loss of grape skin color [25]. Four MYB transcription factors, namely *VvMYBPA1*, *VvMYBPA2*, *VvMYB5a*, and *VvMYB5b*, are known to regulate PA synthesis in grapes [25,26,27]. Additionally, plants possess MYB negative regulatory factors that can suppress a gene or a branch of the flavonoid metabolic pathway. For instance, strawberry *FaMYB1* and petunia *PhMYB27* repress the biosynthesis of anthocyanins and PA [28,29]. *VvMYB4a* and *VvMYB4b* repress general phenylpropanoid biosynthetic genes, and *VvMYBC2-L1* inhibits PA metabolism in grapes [7].

Metabolomic and transcriptomic technologies are commonly used to identify the differential metabolites and understand the mechanisms underlying gene expression regulation in the biosynthetic pathway. By utilizing a combination of transcriptome and metabolome analysis, many reports have detailed the accumulation of anthocyanin and the key genes involved in anthocyanin synthesis in various fruits, including grapes, apples, cherries, and peaches [30,31,32,33,34]. The correlation between color formation and anthocyanin accumulation has been the subject of extensive research in *Vitis vinifera*. However, metabolite profiling of muscadine grapes during berry development has been only investigated by untargeted metabolomics [12]. Advances in metabolomic technology have led to substantial progress in understanding the regulation of flavonoid biosynthesis. Compared with untargeted metabolomics, multi-targeted metabolomics has the advantage of high throughput, high sensitivity, wide coverage, and accurate qualitative and quantitative analysis [32,33]. Thus, to explore the mechanism of skin color changes in muscadine grapes during different development stages, omics methods were employed to detect flavonoids at various developmental stages in the Paulk and Supreme varieties of muscadine grapes. In addition, transcriptomics was utilized to analyze changes in gene expression at these stages. Based on previous research on fruit quality, the total sugar content of Supreme is higher than Paulk. In this research, we further explore the mechanism of flavonoid biosynthesis during berry development. The identification of flavonoids in muscadine grapes and the exploration of the mechanism behind the accumulation of different flavonoids during berry development is essential for future genetic or biotechnological improvements.

## 2. Results

### 2.1. Flavonoid Profiling in the Muscadine Grape During Berry Development

**Figure 1:**
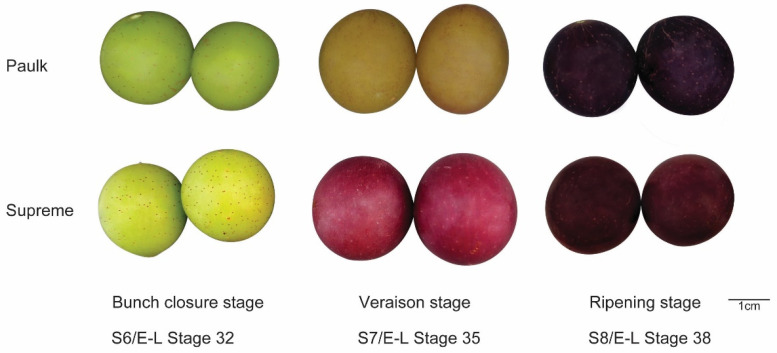
The phenotypes of Paulk and Supreme across three developmental stages.

**Figure 2:**
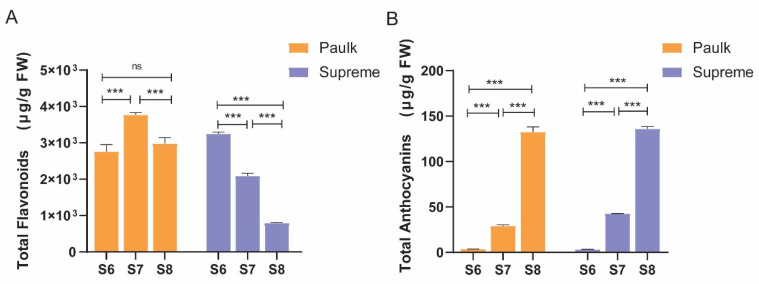
Changes in total flavonoid (**A**) and anthocyanin content (**B**). Bars with the same lowercase letters denote no significant difference, while bars without shared lowercase letters indicate a significant difference. The columns in orange represent Paulk, while the purple bars represent Supreme. The columns represent means ± SEM of three independent experiments. One-way ANOVA was performed for different developmental stages of the same cultivar. *** represents highly significant differences (*p* < 0.001), while ‘ns’ indicates no significant difference.

Two varieties of muscadine grapes were used for analysis. During the bunch closure stage, the peel of both is green. As the fruit develops, the berry color of Paulk transitions to copper and then eventually to black-purple. In contrast, the peel of the Supreme variety changes from green to red and darkens over time. Samples were collected from three developmental stages: bunch closure, veraison stage, and ripening stage (S6, S7, and S8) (Figure 1). A colorimeter was used to measure the color of berries at each stage (Table 1). Total flavonoid and anthocyanin contents were determined in these stages (Figure 2). Two-way ANOVA analysis showed that the variety, development stage, and interaction between the two factors significantly affected color, flavonoid, and anthocyanin contents (Table 2). The results showed that flavonoid contents were lower in the ripening stage in both varieties, consistent with the previous reports by Darwish et al. (2021) [12]. In Paulk, flavonoid contents were higher in the veraison stage, while in Supreme, which is higher in the bunch stage. Anthocyanin contents were increased during berry development in both varieties.

**Figure 3:**
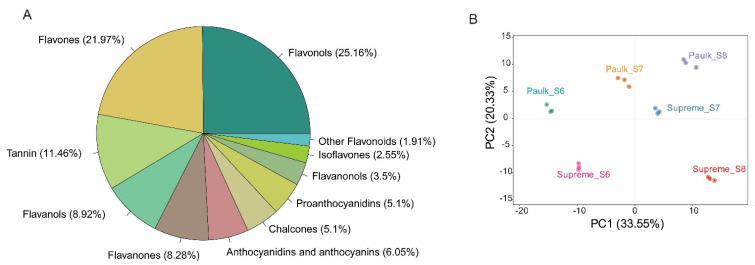
Identification and analysis of flavonoid constituents in muscadine grape berry development. (**A**) Metabolite classification. (**B**) Principal component analysis (PCA) plot of metabolomic samples.

The comprehensive profiling of flavonoids in the two muscadine grapes during berry development was further conducted using a widely targeted metabolomics method. In total, 314 flavonoids were detected, belonging to 11 categories: 16 chalcones, 26 flavanones, 11 flavanonols, 19 anthocyanidins, 69 flavones, 79 flavonols, 28 flavanols, 6 other flavonoids, 36 tannins, 8 isoflavones, and 16 proanthocyanidins (PA) (Table S2) (Figure 3A). The major flavonoid constituents in the muscadine grape were flavone and flavonol, similar to the previous report in *V. vinifera* [35]. In addition, anthocyanidin-di-glucosides are the major class of anthocyanins accumulated in muscadine grapes, such as pelargonidin-3.5-O-diglucosid, cyanidin-3,5-O-diglucoside, and delphinidin-3,5-di-O-glucoside. This study provides valuable insights into the flavonoid composition of muscadine grapes at different development stages.

### 2.2. Differentially Accumulated Metabolites (DAMs) During Muscadine Grape Berry Development

The accumulation of flavonoids exhibited a clear pattern dependent on both variety and developmental stage, as revealed by principal component analysis (PCA). The first component (PC1, 30.48%) separated the bunch closure stage of Supreme and Paulk from the ripening stage of both varieties. PC1 also distinguished the veraison stage of Paulk from Supreme, indicating that PC1 effectively separated colorless stages from colored stages. The second component (PC2, 19.42%) differentiated all development stages of Paulk and the veraison stage of Supreme from the bunch closure and ripening stage of Supreme (Figure 3B).

**Figure 4:**
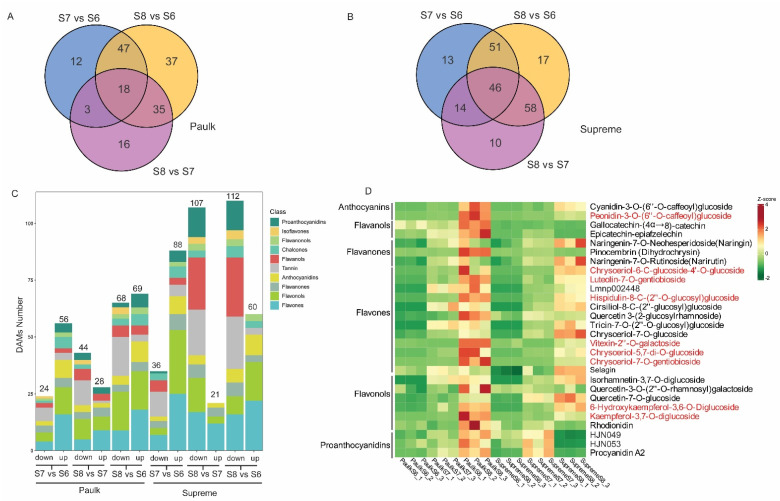
Analysis of differentially accumulated metabolites during various ripening stages. (**A**) Venn diagram illustrating the shared and unique metabolites among the three comparisons of Paulk. Comparisons S7 vs. S6, S8 vs. S7, and S8 vs. S6 revealed 12, 16, and 37 DAMs, respectively, with 18 DAMs detected across all three comparisons. (**B**) Venn diagram illustrating the shared and unique metabolites among the three comparison groups of Supreme. In total, 13, 10, and 17 metabolites were uniquely altered in one comparison, while 46 DAMs were detected across all three comparisons. (**C**) A statistical analysis of the increased and decreased flavonoids across the three comparisons in Paulk and Supreme. The number of DAMs for each comparison group is labeled in the upper column. Different colors were labeled to different metabolites classification. (**D**) Heat map analysis of higher abundant metabolites in the ripening stage of Paulk or Supreme. Metabolites in red font continuously increased during berries development in both two varieties.

To identify the development-stage-dependent flavonoids, a comparative metabolite analysis was conducted for Supreme and Paulk during the berry development. Differentially accumulated metabolites (DAMs) were identified using screening thresholds of |log_2_(fold change)| ≥ 1 and variable importance for the projection (VIP) ≥ 1 in PLS-DA. There are 168 DAMs between different development stages in Paulk, including 45 flavones, 40 flavonols, 26 flavanols, 16 anthocyanins, and 14 PAs (Table S3). Venn diagrams showed that 18 DAMs were shared among the three comparisons (Figure 4A). A comparison between the veraison and bunch closure stage (S7 vs. S6) showed that 24 metabolites decreased and 56 increased in the veraison stage. Meanwhile, comparing the ripening with the veraison stage (S8 vs. S7) showed that 44 and 28 metabolites decreased and increased, respectively. Further analysis of the DAMs categories in Paulk revealed that higher levels of flavone, flavonol, and proanthocyanidin accumulated in the bunch closure stage, while anthocyanidin accumulated in the ripening stage (Figure 4C).

In Supreme, 209 DAMs were detected during berry development (Table S3, Figure 4B). Of these, 88 and 21 DAMs were increased in the comparisons of S7 vs. S6 and S8 vs. S7, respectively, while 36 and 107 DAMs were downregulated in the two comparisons, respectively (Figure 4C). Similar to Paulk, most DAM contents were higher in the veraison stage of Supreme, including flavone, flavonols, and proanthocyanidin. In addition, there are nine DAMs content increased during berries development both in Paulk and Supreme, including peonidin-3-O-(6″-O-caffeoyl) glucoside, chrysoeriol-6-C-glucoside-4′-O-glucoside, luteolin-7-O-gentiobioside, hispidulin-8-C-(2″-O-glucosyl) glucoside, vitexin-2″-O-galactoside, chrysoeriol-5,7-di-O-glucoside, chrysoeriol-7-O-gentiobioside, 6-hydroxykaempferol-3,6-O-diglucoside, kaempferol-3,7-O-diglucoside, which are characteristic compounds in the ripening stage of muscadine grape (Figure 4D).

### 2.3. Differentially Accumulated Metabolites (DAMs) Between Paulk and Supreme

**Figure 5:**
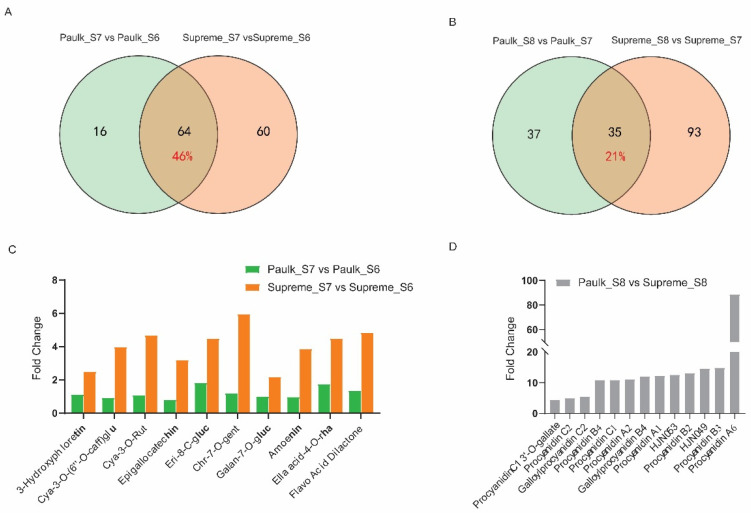
Analysis of differentially accumulated metabolites (DAMs) analysis between the Paulk and Supreme varieties. (**A**) Venn diagram illustrating the shared and unique DAMs in the S7 vs. S6 comparison between Paulk and Supreme. A total of 64 DAMs changed in both varieties, while 60 DAMs and 16 DAMs were changed only in Supreme and Paulk, respectively. (**B**) Venn diagram of the S8 vs. S7 comparison between Paulk and Supreme. A total of 35 DAMs were changed in both varieties, while 93 DAMs and 37 DAMs were changed only in Supreme and Paulk, respectively. (**C**) Ten metabolites were significantly upregulated in the veraison stage of Supreme while unchanged in Paulk. cya, cyanidin; caff, caffeoyl; rut, rutinoside; eri, eriodictyol; chr, chrysoeriol; gent, gentiobioside; gluc, glucoside; ella, ellagic acid. (**D**) Thirteen PAs with higher abundance in the ripening stage of Paulk. HJN049, 2α,3α-Epoxy-5,7,3′,4′-tetrahydroxyflavan-(4β-8-catechin); HJN053, Galloylprocyanidin B4.

To identify the variety-dependent flavonoids, a comparative metabolite analysis was also conducted. A comparison between the veraison and bunch closure stages revealed that 64 DAMs were changed in both varieties, accounting for 45.7% of all DAMs (Figure 5A). Among these commonly changed metabolites, 16 DAMs (25%) decreased, while 48 DAMs (75%) increased during the veraison stage. Fourteen of the top twenty-fold change metabolites in Paulk and Supreme were the same, such as cirsiliol-8-C-(2″-glucosyl)-glucoside, vitexin-2″-O-galactoside, chrysoeriol-5,7-di-O-glucoside, isorhamnetin-3-O-sophoroside, petunidin-3,5-di-O-glucoside, hispidulin-8-C-(2″-o-glucosyl)-glucoside, isorhamnetin-3,7-O-diglucoside, cyanidin-3-O-glucoside chloride, tricin-7-O-(2″-O-glucosyl)-glucoside, chrysoeriol-6-C-glucoside-4′-O-glucoside (Figure S1). These may be the main flavonoids in muscadine grapes and play significant roles during berry development. There were 60 and 16 DAMs specifically changed in Supreme and Paulk, respectively (Figure 5A). Among the specifically changed metabolites, such as cyanidin-3-O-(6″-O-caffeoyl)-glucoside, cyanidin-3-O-rutinoside, epigallocatechin, eriodictyol-8-C-glucoside, chrysoeriol-7-O-gentiobioside were only increased in the veraison stage of Supreme, which may be related to the different color in veraison stage between Paulk and Supreme (Figure 5C, Table S4).

In the comparison between the ripening and veraison stage, only 35 DAMs were changed in both varieties, accounting for 21.2% of all DAMs, while 93 and 37 DAMs were specifically changed in Supreme and Paulk (Figure 5B). Differences in flavonoid accumulation between the two varieties were mainly observed in the late development stage. Contrasting with the comparison between the bunch closure and veraison stages, only one metabolite, vitexin-2′-O-galactoside, was common in the top twenty-fold changed metabolites between Paulk and Supreme during the late development stage (Figure S1). This metabolite may serve as a development biomarker in the growth stage of *V. rotundifolia*. Interestingly, 13 proanthocyanidins, including procyanidin C2, procyanidin B4, and procyanidin A2, accumulated higher levels in the ripening stage of Paulk compared to Supreme, which may be related to the color differences between the two varieties (Figure 5D, Table S4).

### 2.4. Transcriptome Analysis of Muscadine Grape During Development

To investigate the potential molecular mechanisms of flavonoid biosynthesis during berry development, RNA-seq analyses were performed on 18 samples. These 18 libraries generated 163.39 G of clean data, which have been deposited in the NCBI database (www.ncbi.nlm.nih.gov/bioproject/PRJNA1039948 (accessed on 14 December 2024)). Following read filtering and adaptor sequence trimming, an average of 60,518,902 reads were obtained. The Q30 percentages, QC20 percentages, and GC percentages were all high, exceeding 92%, 97%, and 45%, respectively (Table S5). For the 18 libraries, approximately 76.18% of the clean reads were successfully mapped to the *V. vinifera* reference genome sequence. More than 21,644 genes were annotated in various databases, including GO, KEGG, KOG, NR, Pram, Swissprot, and Tremble database.

### 2.5. Differentially Expressed Gene Analysis

**Figure 6:**
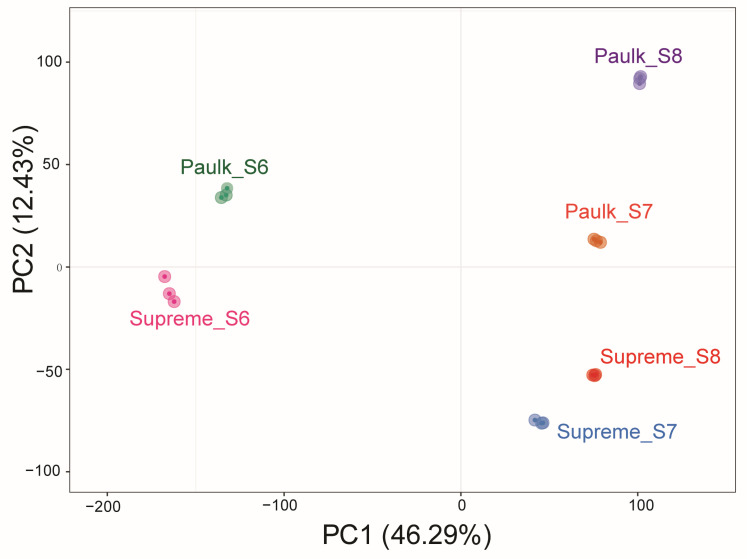
Principal component analysis (PCA) of transcriptome data in muscadine grapes.

In the PCA, the first component (PC1) distinguished the bunch closure stage from the other stages, while the second component (PC2) separated the Paulk variety from Supreme. This analysis also demonstrated excellent biological replicates across all samples (Figure 6). To further study changes in gene expression profiles during berry development, differentially expressed genes (DEGs) were screened under the criteria of |log_2_Fold Change| ≥ 1 and FDR < 0.05. A heatmap depicting the hierarchical clustering of all DEGs showed that the samples were divided into three groups according to developmental stages. The highest expression levels of most genes were observed in the bunch closure stage in both varieties, followed by the veraison stage of Paulk (Figure S2).

**Figure 7:**
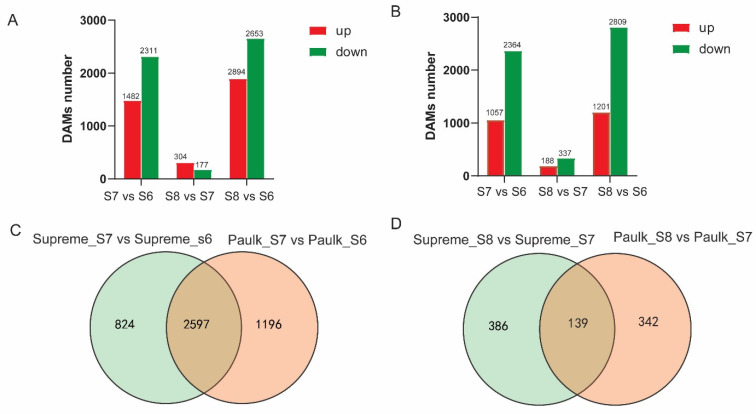
Statistics and Venn diagram of differentially expressed genes (DEGs) in Paulk and Supreme. (**A**) Statistics of upregulated and downregulated genes in the three comparison groups in Paulk. (**B**) Statistics of upregulated and downregulated genes in the three comparison groups in Supreme. (**C**) Venn diagram illustrating the shared and unique DEGs in the S8 vs. S7 comparison between Paulk and Supreme. (**D**) Statistics of upregulated and downregulated genes in the three comparison groups in Supreme.

In Paulk, there were 5066 genes differentially expressed during berry development, with 135 genes detected among all three stages (Figure S3 and Table S6). Specifically, 1482, 304, and 1894 genes’ expression levels were increased, while 2311, 177, and 2653 genes decreased in the comparisons of S7 vs. S6, S8 vs. S7, and S8 vs. S6, respectively (Figure 7A). The DEG numbers in the S7 vs. S6 comparison were more than those in the S8 vs. S7 comparison, and more than half of the DEGs were downregulated in the veraison stage compared with the bunch closure stage. In Supreme, 4010 genes were differentially expressed during fruit ripening, with 153 genes detected across the three stages (Figure S3 and Table S6). Specifically, 1057, 188, and 1201 DEGs increased, while 2364, 337, and 2809 genes significantly reduced in the three comparisons (Figure 7B).

When comparing Paulk and Supreme, 2597 (56.2%) DEGs were the same in the S7 vs. S6 comparison, while 139 DEGs (16%) were the same in the S8 vs. S7 comparison between the two varieties. This showed that the difference in the S8 vs. S7 comparison between Paulk and Supreme was more significant than that of S7 vs. S6 (Figure 7C,D).

To further explore the developmental mechanisms, the identified DEGs were subjected to KEGG pathway enrichment analysis. The top 20 significantly enriched metabolic pathways in Paulk and Supreme are shown in Figure S4. The biosynthesis of secondary metabolites and phenylpropanoid biosynthesis were the dominant enriched pathways in the S7 vs. S6 comparison, while flavone and flavonol biosynthesis were significantly enriched in the S8 vs. S7 comparison in both varieties. Additionally, plant hormone signal transduction and starch and sucrose metabolism pathways were also enriched in the S7 vs. S6 comparison.

### 2.6. Candidate Genes Involved in Flavonoid Biosynthesis in V. rotundifolia

**Figure 8:**
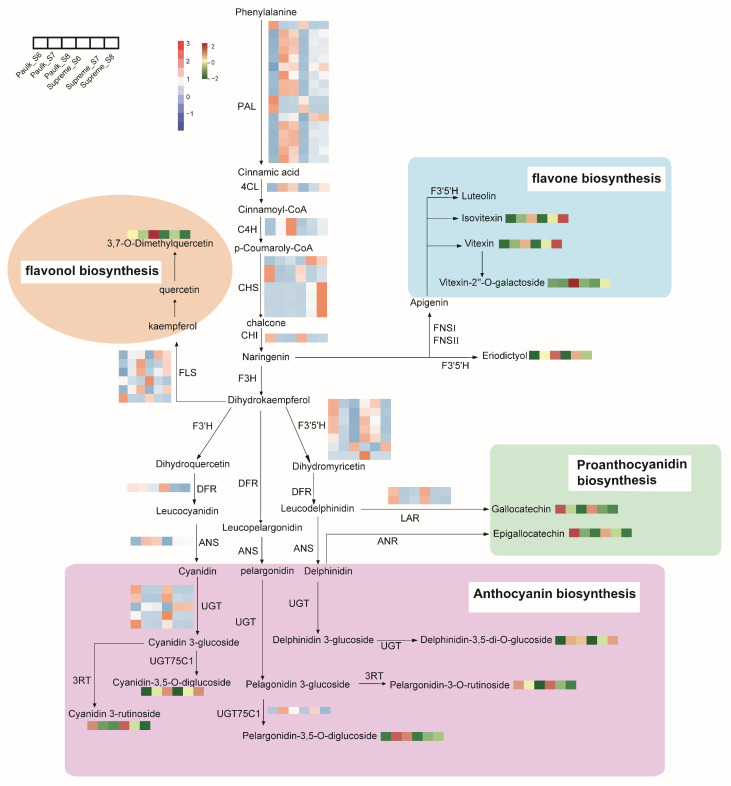
Integrated pathways of differentially accumulated metabolites (DAMs) and differentially expressed genes (DEGs) involved in flavonoid biosynthesis. The pathway was reconstructed based on k00941, k00942, k00943, and k00944 in the KEGG database. Red and blue colors denote upregulated and downregulated genes, respectively. The red and green colors represent high and low relative contents of flavonoids, respectively. Key genes include PAL (phenylalanine ammonia-lyase), 4CL (4-coumarate CoA ligase), DFR (flavanone 4-reductase), CHS (chalcone synthase), FLS (flavonol synthase), F3H (flavanone 3-dioxygenase), CHI (chalcone-flavonone isomerase), anthocyanidin 3-O-glucosyltransferase, LAR (leucoanthocyanidin reductase), C4H (trans-cinnamate 4-monooxygenase), and F3′H (flavonoid 3′-hydroxylase), ANS (anthocyanidin synthase), F3′5′H (flavonoid 3′,5′-hydroxylase), and anthocyanidin 3-O-glucosyltransferase.

To explore the flavonoid biosynthesis pathway during berry development in *V. rotundifolia*, we examined the flavonoid biosynthesis in muscadine grapes (Figure 8). Based on the KEGG pathways and Gene Ontology function analysis, 168 DEGs and 24 DAMs were enriched in Paulk. In comparison, 159 DEGs and 35 DAMs were enriched in Supreme (Table S7). To identify genes involved in the flavonoid biosynthesis pathway of the muscadine grape, we detected the expression patterns of structural genes and transcriptors. Phenylalanine is first catalyzed to synthesize coumaryl-CoA through the action of three enzymes: phenylalanine ammonia lyase (PAL), 4-coumarate CoA ligase (4CL), and cinnamic acid 4-hydroxylase (C4H). Most *PAL*, *4CL*, and *C4H* genes exhibited low expression in S6, and their expression levels significantly increased in S7 and S8 in both varieties, suggesting that high expression of these upstream genes of the phenylpropane metabolic pathway promotes the synthesis of more second metabolites in the veraison and ripening stages, especially in Paulk. Chalcone synthase (CHS) and chalcone isomerase (CHI) mediate the reaction from coumaroyl-CoA to naringenin. Three *CHS* genes (*VIT_16s0022g01020*, *VIT_16s0022g01140*, and *VIT_16s0022g01190*) were upregulated only 3–4 fold in Supreme, which may be related to specifically accumulated flavones in the ripening stage of Supreme, such as chrysoeriol-7-O-glucoside. Flavonol synthase (FLS) is a key enzyme on the flavonol branch. Four *FLS* genes (*VIT_08s0105g00380*, *VIT_11s0118g00370*, *VIT_11s0118g00390*, and *VIT_18s0001g03470*) were upregulated, which may relate to the higher levels of flavonols in the ripening stage.

Flavonoid 3′5′-hydroxylase (F3′5′H) participates in catalyzing the hydroxylation of naringenin and dihydrokaempferol. The transcript of the *F3′5′H* gene (*VIT_08s0007g05160*) was increased during the berry development of the two varieties, which may be related to the higher accumulation of eriodictyol. Dihydroflavonols were catalyzed by dihydroflavonol 4-reductase (DFR) to produce leucoanthocyanidin, then further catalyzed by anthocyanidin synthase (ANS) to produce anthocyanidins. Additionally, leucoanthocyanin reductase (LAR) mediates the reaction from leucoanthocyanin to flavanol, a starting unit for PA polymers. Most *F3′5′H* and *LAR* genes downregulated during berry development, possibly related to lower gallocatechin content in the ripening stage. Compared to Supreme, *DFR* (*VIT_16s000039g02350*) and *LAR* (*VIT_17s000g04150*) showed 3–4 fold higher expression levels in the ripening stage of Paulk, suggesting that the two genes may related to the upregulated of PA in Paulk. The transcription level of *ANS* (*VIT_02s0025g04720*) was upregulated 4–5 fold in the two varieties at the ripening stage, which may be related to the accumulation of anthocyanidins. In addition, UDP-glucose:flavonoid-3-O-glucosylltransferase (UGT) and anthocyanidin-3-glucoside rhamnosyltransferase (3RT) involved in the glycosyl modification of anthocyanidins. UGT75C1 and 3RT competed for the same substrate for anthocyanidin 3-glucoside. Then, upregulated *UGT75C1* (*VIT_05s0062g00720*) drives the synthesis of more anthocyanidin 3,5-O-glucoside and less anthocyanidin-3-O-rutinoside during the veraison stage of Paulk and Supreme.

To further clarify the relationship between these candidate genes and DAMs, the correlation between them was analyzed. *4CL* (*VIT_01s0010g03720*) and *MYBA1* (*VIT_02s0033g00410*) were significantly positively correlated with anthocyanin 3,5-O-diglucoside, while *CHS* (*VIT_16s0022g01020*) was negatively correlated with procyanidin (Table S8).

**Figure 9:**
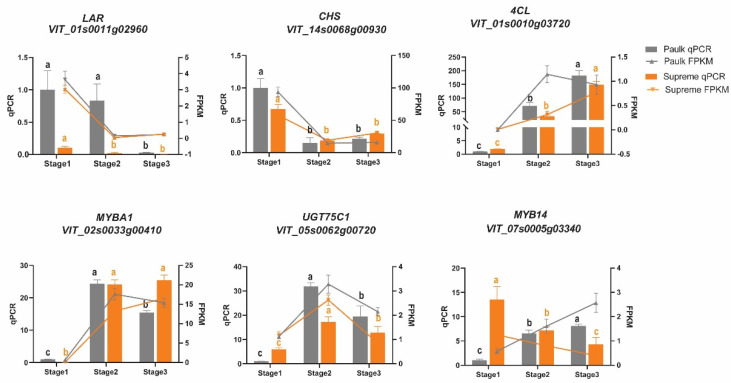
Expression level analysis of flavonoid-biosynthesis-related genes. Quantitative real-time polymerase chain reaction analysis results are displayed in bar charts, and the FPKM values based on transcriptome are displayed in line charts. The columns and lines in gray represent Paulk, while the orange bars and lines represent Supreme. The gene expression levels of each variety at different stages were assessed using one-way ANOVA (*p* < 0.05). Data are presented as the means ± standard deviation. Bars with the same lowercase letters denote no significant difference, while bars without shared lowercase letters indicate a significant difference; ns stands for no statistical difference.

Subsequently, to confirm the candidate structural genes and TFs involved in the biosynthesis of flavonoids, we performed RT-qPCR analysis for genes based on transcriptome (Figure 9). As shown in Figure 9, we found that the expression patterns of nine randomly selected genes were highly accordant with the results from RNA-seq, with the exception of *LAR* and *4CL* in Paulk.

## 3. Discussion

The quality of fruit, including aspects such as flavor and color, as well as its health benefits, is influenced by its phytochemical composition. To identify the flavonoid profile of muscadine grapes grown in Hubei and to investigate the mechanisms behind flavonoid accumulation during berry development, we analyzed two types of muscadine grapes with different berry colors using transcriptomics and metabolomics. Considering the impact of sampling time and position on omics data, we carried out preliminary work and sampled at the three main time points to explore the mechanism of flavonoid biosynthesis and find the mechanism of skin coloring during berry development in this manuscript.

In this study, we identified six types of anthocyanins, which were predominantly present in the form of 3,5-O-diglucoside anthocyanins in muscadine grapes. During berry development of the Supreme and Paulk, there was an increase in pelargonidin 3,5-O-diglucoside, cyanindin-3,5-O-diglucoside, and delphinidin-3,5-di-O-glucoside. This result is a departure from the anthocyanin components found in *V. vinifera* but aligns with previous reports in muscadine grape [36,37]. When comparing the anthocyanin components between the two varieties, Paulk was found to be enriched in cyanidin-3-O-(6″-O-p-coumaroyl) glucoside and pelargonidin-3,5-O-diglucoside during the ripening stage, while Supreme produced more malvidin-3-O-rutinoside-5-O-glucoside during the veraison stage, which may be related to a higher expression of F3′5′H in it. In both varieties, peonidin-3-O-(6″-O-caffeoyl) glucoside accumulated in the ripening stage. In addition, it should be noted that the whole berries except seeds were used for the analysis, which may influence the composition of anthocyanins in the peel. Furthermore, though the two varieties have similar levels of soluble solids and fruit size, the absence of dry weight normalization in the current analysis could still introduce potential variability in the results.

In the early stage of flavonoid biosynthesis, the *PAL*, *4CL*, *C4H*, and *CHS* genes exhibited varying expression levels between the Paulk and Supreme varieties. We identified 18 genes encoding phenylalanine ammonia-lyase, with 15 showing higher expression during the veraison and ripening stages in muscadine grapes, particularly in Paulk. Phenylalanine ammonia-lyase, which catalyzes the initial step of the phenylpropanoid pathway, leads to an increased production of second metabolites due to the high expression levels of these genes [38]. Seven *CHS* genes displayed differential expression during berry development, with five upregulated exclusively in Supreme and the remaining two downregulated in both varieties. Previous research has indicated a relation between the expression level of the *CHS* gene and anthocyanins in grape berry skins and purple pigmentation in wheat [39,40]. The upregulated expression of *CHS* in Supreme may be the primary reason for the higher accumulation of chrysoeriol-7-O-glucoside. Furthermore, CHS synthesizes phloretin from dihydro-4-coumaroyl-CoA, and consistent with the elevated expression levels of *CHS*, phloretin was found to be enriched in Supreme. F3′5′H, which catalyzes the conversion of dihydrokampferol to dihydroquercetin and subsequently forms delphinidin or cyanidin catalyzed by two enzymes (DFR and ANS) [41]. It has been reported that a high expression level of *F3*′*5*′*H* aligns with the overaccumulation of delphinidin-based anthocyanins (blue color), while *F3*′*H* expression is related to cyanidin-based anthocyanins (red-orange) [42,43,44,45]. A higher level of *F3*′*5*′*H* results in a higher level of delphinidin-based anthocyanins. In this study, the *F3*′*5*′*H* (*VIT_08S0007g05160*) gene was significantly upregulated (Figure 9), which may play a major role in delphinidin.

MYB, one of the largest transcription factor families in plants, plays a significant role in regulating flavonoid biosynthesis by interacting with *bHLHs* and *WDR* to form the MBW protein complex [35,46]. Seven *MYBs*, namely *MYBA1*, *MYBA2*, *MYB5a*, *MYB5b*, *MYBPA1*, *MYBPA2,* and *MYBC2-L1*, have been reported to be associated with the regulation of flavonoid biosynthesis in grapes [30,32,34,47,48,49]. Here, 66 MYBs were screened from the differentially expressed genes (DEGs). In the heatmap, 43 MYBs were shown higher expression levels (Figure S4). During the development of muscadine grape berries, the expression levels of *VvMYBCS1* (MYB5a) and *MYBA1* were upregulated, contributing to the accumulation and regulation of anthocyanin biosynthesis [50]. Our findings revealed that *VvMYBA1* (*VIT_02s0033g00410*) was significantly highly expressed during the veraison and ripening stages in both varieties (Figure 9), aligning with previous reports [25]. Recently, *VvMYB14* has been implicated in melatonin-induced PA biosynthesis in grape seeds [51]. Interestingly, in this study, *MYB14* (*VIT_07s0005g03340*) increased 8-fold in Paulk, while it decreased 3.1-fold in Supreme (Figure 9), indicating its potential role in the higher accumulation of PA in Paulk.

*Glutathione S-transferases* (GSTs) and *multidrug and toxic extrusion* (MATE) transporters play crucial roles in the vacuolar accumulation of flavonoids in plants. Five GST genes have been previously implicated in the vacuolar sequestration of anthocyanins [52]. In this study, three GST genes, including *GST4* (*VIT_04s0079g00690*)*, GST3* (*VIT_12s0028g00920*), and *GST2* (*VIT_07s0005g00030*), showed differential expression levels during berry development. Two additional GST candidate genes, *VIT_19s0093g00190* and *VIT_19s0015g02680*, were also significantly upregulated during berry development (Table S7), suggesting their potential involvement in anthocyanin accumulation in muscadine grapes. Furthermore, based on gene KEGG annotation, we identified 97 MATE genes from DEGs. By considering the FPKM values of gene expression, six genes showed specifically higher expression levels in the veraison stage of Supreme (Figure S5). Subsequently, correlation analysis between *MATE* genes and DAMs revealed that *VIT_18s0001g06790* exhibited a significant correlation with flavanols (epicatechin-3′-O-β-D-glucopyranoside, epicatechin-4′-O-β-D-glucopyranoside, epicatechin glucoside, and catechin-4-β-D-galactopyranoside) (Table S8). These four metabolites, prerequisite material for the synthesis of proanthocyanins, showed indeed elevated levels in the veraison stage of Supreme, suggesting that *VIT_18s0001g06790* may be a key MATE gene involved in muscadine grape proanthocyanin transportation. However, due to the potential impact of large-scale differentially expressed genes (DEGs) on the analytical accuracy of DEseq2, we will conduct consensus analysis, such as using edgeR to filter DEGs across these methods; meanwhile, we will perform RT-qPCR validation.

## 4. Materials and Methods

### 4.1. Plant Materials

Berries of muscadine grapes were collected from the vineyard of the Xianning Academy of Agricultural Sciences in Hubei, China. Three-year-old ‘Paulk’ and ‘Supreme’ grapes with similar growth vigors and sizes were selected as the experimental materials. The vine rows were oriented from north to south and spaced at 4.0 m. The space between vines was 2.0 m, and the frame type was T. Both the Paulk and Supreme varieties were well-watered and from pests and disease. The growth of the grapevine is classified into 47 stages, including 8 major stages, according to the modified E-L system [7,53]. Samples were collected at three developmental stages: bunch closure (S6/E-L 32 stage), veraison (S7/E-L 35 stage), and ripe (S8/E-L 38 stage). Veraison was determined as the time when the berries began to soften, and ~50% of the berries started to change color. At the same time, detecting soluble solids content and observing seed color were two criteria to determine berry ripeness. Berries at the same developmental stage from three different clusters and different vines were combined to form one sample, with three biological replicates taken at each stage. The color parameters L* (lightness), a* (green-red coordinate), and b* (blue-yellow coordinate) were measured using a Minolta Chroma Meter CR-400 colorimeter (Konica Minolta Sensing Europe B.V., Osaka, Japan). Samples used for omics analysis were immediately frozen in liquid nitrogen and stored at −80 °C for future analysis.

### 4.2. Metabolite Extraction

Metabolomic analysis was conducted using an ultra-performance liquid chromatography–electrospray ionization–tandem mass spectrometry (UPLC-ESI-MS/MS) system (AB Sciex, Framingham, MA, USA). Freeze-dried samples except for seeds were ground into powder using a mixer mill and metabolites were extracted from 100 mg of this powder using 1.0 mL of a 70% aqueous methanol solution overnight at 4 °C. A concentration of 1 mg/L of 2-chlorophenylalanine was used as internal standard. The extracted solution was then filtered through a 0.22 μM microporous membrane and injected into LC-MS vials. In addition, the quantification of total flavonoid and anthocyanin contents was determined by the biochemical kits (Norminkoda Biotechnology Co., Ltd., Wuhan, China), following the manufacturer’s instructions.

### 4.3. Metabolic Profiling of Flavonoids

Identification and quantification of flavonoids were performed by MetWare Biotechnology Co. Ltd. (Wuhan, China) using multiple reaction monitoring (MRM) technology [54]. Precisely, 5 μL of sample extracts were injected and analyzed using a Shim-pack UFLC SHIMADZU CBM30A system (Shimadzu Co., Kyoto, Japan). A Waters ACQUITY UPLC HSS T3 C18 (Waters Co., Milford, MA, USA) column was used for this process. The chromatographic separation conditions were as follows: the column oven temperature was set at 40 °C; the solvent system consisted of A (0.04% formic acid): B (acetonitrile with 0.04% formic acid); and the flow rate was set at 0.4 mL/min. The eluting gradient program was set to 0–11 min, 95–5% A; 12 min, 5% A; 12–12.1 min, 5–95% A; and 12.1–15 min, 95% A. The effluent was connected to a triple quadrupole–linear ion trap mass spectrometer (AB4500 Q TRAP system) (AB Sciex, Framingham, MA, USA) and controlled by Analyst 1.6.3 software (Sciex, Fort Collins, NWT, Canada). A QC sample, prepared by mixing aliquots of all sample extracts, was injected after every set of 10 samples to monitor technical reproducibility. Metabolite identification was performed using a self-built Metware database in conjunction with the public databases.

### 4.4. Transcriptome Sequencing and Annotation

A total of 18 samples were collected from the Paulk and Supreme, encompassing three developmental stages. For each sample, 1 g of powdered material was used for RNA extraction using the RNA Easy Fast Plant Extraction Kit (TIANGEN, Beijing, China) as per the manufacturer’s instructions. The purity and quantification of RNA were assessed using a NanoDrop spectrophotometer, and RNA integrity was further evaluated using an Agilent 2100 bioanalyzer system (Agilent Technology, Santa Clara, CA, USA). High-quality RNA samples were used to construct the libraries. The quality of these libraries was measured using the Qubit RNA assay kit in conjunction with a Qubit 2.0 fluorometer (Life Technology, Carlsbad, CA, USA) [55]. The libraries were then sequenced on the Illumina sequencing platform, yielding paired-end reads. The raw reads were initially filtered using Fastp to remove adapters and low-quality sequences, resulting in clean reads [56]. *The V. vinifera* genome sequence (http://ftp.ensemblgenomes.org/pub/plants/release-52/fasta/ (accessed on 17 October 2023)) served as a reference for aligning the filtered reads, which was performed using HISAT2 [57]. The functional annotation of genes was carried out using several databases, including NCBI non-redundant (NR), Kyoto Encyclopedia of Genes and Genomes (KEGG), Eukaryotic Clusters of Orthologous Groups (KOG), Swiss PROT sequence protein (Swissprot), Gene Ontology (GO), and the homologous protein family (Pfam).

### 4.5. Data Analysis

Hierarchical clustering analysis (HCA) and principal component analysis (PCA) (PLS-DA) were performed using Metware Cloud, a free online platform for data analysis (https://cloud.metware.cn (accessed on 12 March 2025)). ANOVA was used in this study with a significant level of *p* ≤ 0.05. The fragments per kilobase of transcript per million mapped reads (FPKM) were used for differential expression analysis, which was conducted using the edgeR 3.24.3 package using Metware Cloud [58]. The thresholds for this analysis were |log_2_(fold change)| ≥ 2 and a false discovery rate (FDR) < 0.05. GO function enrichment and KEGG pathway enrichment analysis of DEGs were performed with Goseq and KOBAS [59,60]. Finally, Pearson correlation coefficients among the DEGs and DAMs were analyzed using R version 3.5.1 in Metware Cloud [61]. Strong correlations were screened based on the threshold of |coefficients| ≥ 0.8 and *p* < 0.05.

Data were analyzed using analysis of variance (ANOVA) in SPSS 26.0 software (SPSS Inc., Chicago, IL, USA). For one-way analysis of variance, three complementary post hoc multiple comparisons were performed using LSD, Tukey’s b, and Waller–Duncan tests to verify the consistency of intergroup differences. A *p*-value < 0.05 was considered statistically significant.

### 4.6. RT-qPCR Analysis

To validate the accuracy of RNAseq data, five structural genes implicated in flavonoid biosynthesis were selected. Complementary DNA (cDNA) was synthesized using a PrimeScript RT Reagent Kit (TakaRa Co., Ltd., Osaka, Japan). Actin served as the reference gene, and the primers utilized are detailed in Table S1. RT-qPCR analyses were conducted following the methodology outlined in our previous report [62]. A one-way ANOVA was employed to determine significant differences.

## 5. Conclusions

The color of berry skin is a significant characteristic trait that influences fruit’s appeal to consumers. The mechanisms behind berry coloration during the growth of muscadine grapes were revealed using metabolomics and transcriptomics techniques. The total anthocyanin content increases during fruit maturation. Anthocyanidin-di-O-glucoside is the primary anthocyanin derivative in *V. rotundifolia*, which is different from *V. vinfera*. The majority of flavones, flavanols, and flavonols had higher concentrations at the veraison stage. The PA content also affects the color difference between the two varieties. Furthermore, genes involved in flavonoid biosynthesis, regulation, and transport were identified. The expression levels of most DEG were higher in the bunch closure stage in both varieties. The upregulation of PAL, C4H, and 4CL promotes the synthesis of more flavonoids during the veraison and ripening stages. Genes involved in the anthocyanin and PA biosynthesis were also identified. This study provides comprehensive insights into flavonoid metabolism and berry coloration in *Vitis rotundifolia*.

## Figures and Tables

**Table 1:** The colorimetric parameters L*, a*, and b* of each stage.

Parameters	Paulk			Supreme		
	Stage 1	Stage 2	Stage 3	Stage 1	Stage 2	Stage 3
L*	60.85 ± 0.71 a	41.04 ± 0.56 b	25.90 ± 0.38 c	54.93 ± 0.45 a	34.00 ± 1.18 b	26.10 ± 0.45 c
a*	−11.00 ± 1.21 b	5.03 ± 1.47 a	4.75 ± 1.31 a	−8.48 ± 1.57 c	16.8686 ± 2.78 a	4.84 ± 1.57 b
b*	31.24 ± 1.51 a	10.43 ± 1.97 b	1.02 ± 0.28 c	25.48 ± 0.36 a	5.73 ± 0.88 b	1.16 ± 0.36 c

Note: L* is the luminance or lightness component. The a* (from green to red) and b* (from blue to yellow) are two chromatic components. Differences in every parameter among the three development stages of each variety were analyzed by one-way ANOVA. Different letters (a, b, c) indicate significant differences (*p* < 0.05).

**Table 2:** Results of two-way ANOVA analysis on the effects of variety and development stage on color parameters, flavonoids, and anthocyanin contents.

Source of Variance	df	Flavonoid Content	Anthocyanin Content	Colorimetric Parameters L*	Colorimetric Parameters a*	Colorimetric Parameters b*
Variety (D)	1	8.59 × 10^−11^ **	0.02 *	8.75 × 10^−14^ **	5.88 × 10^−08^ **	2.77 × 10^−08^ **
Development stage (S)	2	1.23 × 10^−09^ **	6.11 × 10^−15^ **	1.05 × 10^−34^ **	9.26 × 10^−20^ **	2.16 × 10^−28^ **
DÍS	2	2.65 × 10^−10^ **	0.05 *	4.60 × 10^−11^ **	3.35 × 10^−8^ **	7.67 × 10^−6^ **

Note: The asterisks represent statistically significant differences at *p* < 0.05 (*) and *p* < 0.01 (**).

## Data Availability

The original contributions presented in this study are included in the article/Supplementary Material. Further inquiries can be directed to the corresponding author(s).

## Supplementary Materials

The following supporting information can be downloaded at https://www.mdpi.com/article/10.3390/plants14132025/s1: Figure S1. The top twenty-fold changed metabolites in different comparison groups. Figure S2. Clustering analysis of transcriptome data. Heat map illustrating all genes from muscadine grapes. Figure S3. Venn diagram depicting the shared and specific DEGs between the comparison groups. Figure S4. Clustering analysis of differentially accumulated MYB transcription factors in muscadine grapes. Figure S5. Clustering analysis of differentially accumulated MATE genes in Supreme. Table S1. RT-qPCR primers used in this study. Table S2. List of all flavonoids identified by LC-ESI-MS/MS in all samples. Table S3. List of DAMs detected during berry development in Paulk and Supreme. Table S4. List of DAMs in comparison of development stages between Paulk and Supreme. Table S5. Summary of RNA-seq in different muscadine grape samples. Table S6. List of DEGs identified during berry development in Paulk and Supreme. Table S7. List of flavonoid-accumulation-related genes in Paulk and Supreme. Table S8. The Pearson correlation coefficient of differentially accumulated metabolites and MATE genes.
