# Supplementary material for: Transcriptome and Metabolome Analyses of Flavonoid Biosynthesis During Berry Development of Muscadine Grape (Vitis rotundifolia Michx)
Source: Plants (Basel). 2025 Jul 2;14(13):2025. doi: 10.3390/plants14132025 (PMC12252241; doi:10.3390/plants14132025)
Supplement: Supplementary file 1 [file plants-14-02025-s001.zip › supplementary data/Table S1 RT-qPCR primers.docx]

Table S1: Real-time primers

| Gene |  | sequence（5‘-3’） | product size |
| --- | --- | --- | --- |
| VIT_01s0011g02960 | F | GAACTCGCATCTGTGTGGGA | 101bp |
|  | R | GGGATGATGTTTTCTCCGGC |  |
| VIT_14s0068g00930 | F | GAAGTTCAAGCGCATGTGTGA | 123bp |
|  | R | TGACGGGCATCAAGAGATGG |  |
| VIT_08s0105g00380 | F | TGGGGATCAGATTCAGGTGATA | 164bp |
|  | R | CTGGTCGATCCTTGCTCACA |  |
| VIT_19s0014g04980 | F | TCCAGAATGGGGTCATTGGC | 188bp |
|  | R | TCTTCCTCCACCAACCTTTGAA |  |
| VIT_16s0039g01110 | F | CGAGCAGTGAGTGGGTGAT | 147bp |
|  | R | ATATCCCAGCATTCAAGAACCTGAT |  |
| VIT_16s0022g01020 | F | CGTAGGTGCTCGTGTTCTTGT | 184bp |
|  | R | TGCGGAGATGAGCTGGAATAAT |  |
| VIT_01s0010g03720 | F | TGTCCAAGTCCAAGAGGCAT | 170bp |
|  | R | TCTTGGGGAGGGAGATACCG |  |
| VIT_11s0078g00290 | F | TTCATACAGGCAACGCGG | 199bp |
|  | R | CATTAGCAGCCATTATTTCCCTTC |  |
| VIT_05s0062g00720 | F | TCAAAACCAGACGGCTCAGT | 166bp |
|  | R | TGCTGTTGTTCGTCATCTCCT |  |
| VIT_08s0007g05160 | F | GGAGCCGAGATTGGACACAT | 161bp |
|  | R | TCATTAGACTCCGACCCCTGT |  |
| VIT_02s0033g00410 | F | AATTTGTTGGGGAACAGATGGT | 104bp |
|  | R | ACCTCCTTTTTGAAGTGGTGAC |  |
| VIT_07s0005g03340 | F | AAGGCTGGGCAATAGATGGTC | 137bp |
|  | R | AGTGTCTTTTAGGGGGTGGC |  |
| actin-7 | F | AGCTGGAAACTGCAAAGAGCAG | 95bp |
|  | R | ACAACGGAATCTCTCAGCTCCA |  |
