# Supplementary figures and images for: Transcriptome and Metabolome Analyses of Flavonoid Biosynthesis During Berry Development of Muscadine Grape (Vitis rotundifolia Michx)
Source: Plants (Basel). 2025 Jul 2;14(13):2025. doi: 10.3390/plants14132025 (PMC12252241; doi:10.3390/plants14132025)

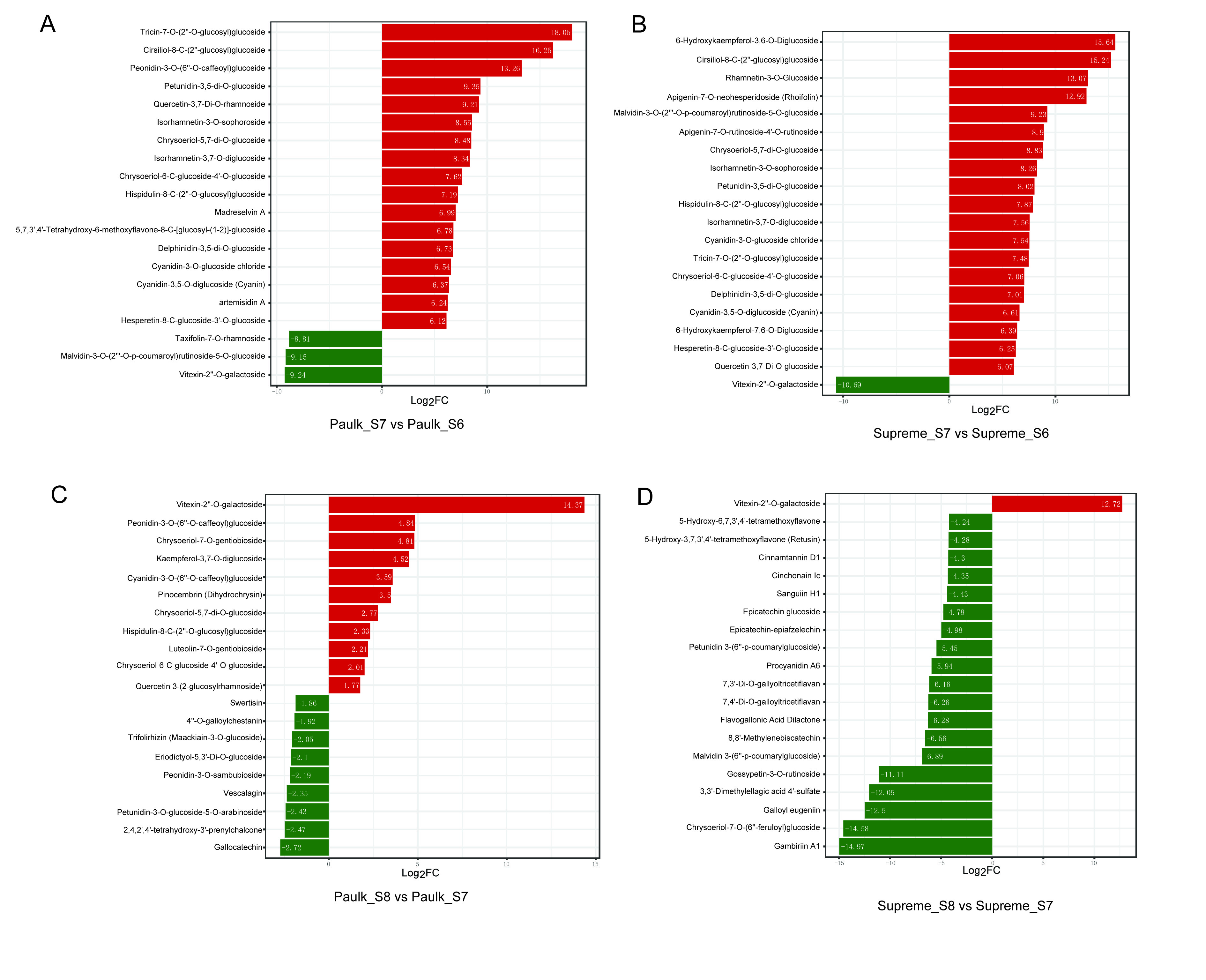

Supplement: Supplementary file 1 [file plants-14-02025-s001.zip › supplementary data/Fig S1 top metabolites.jpg]

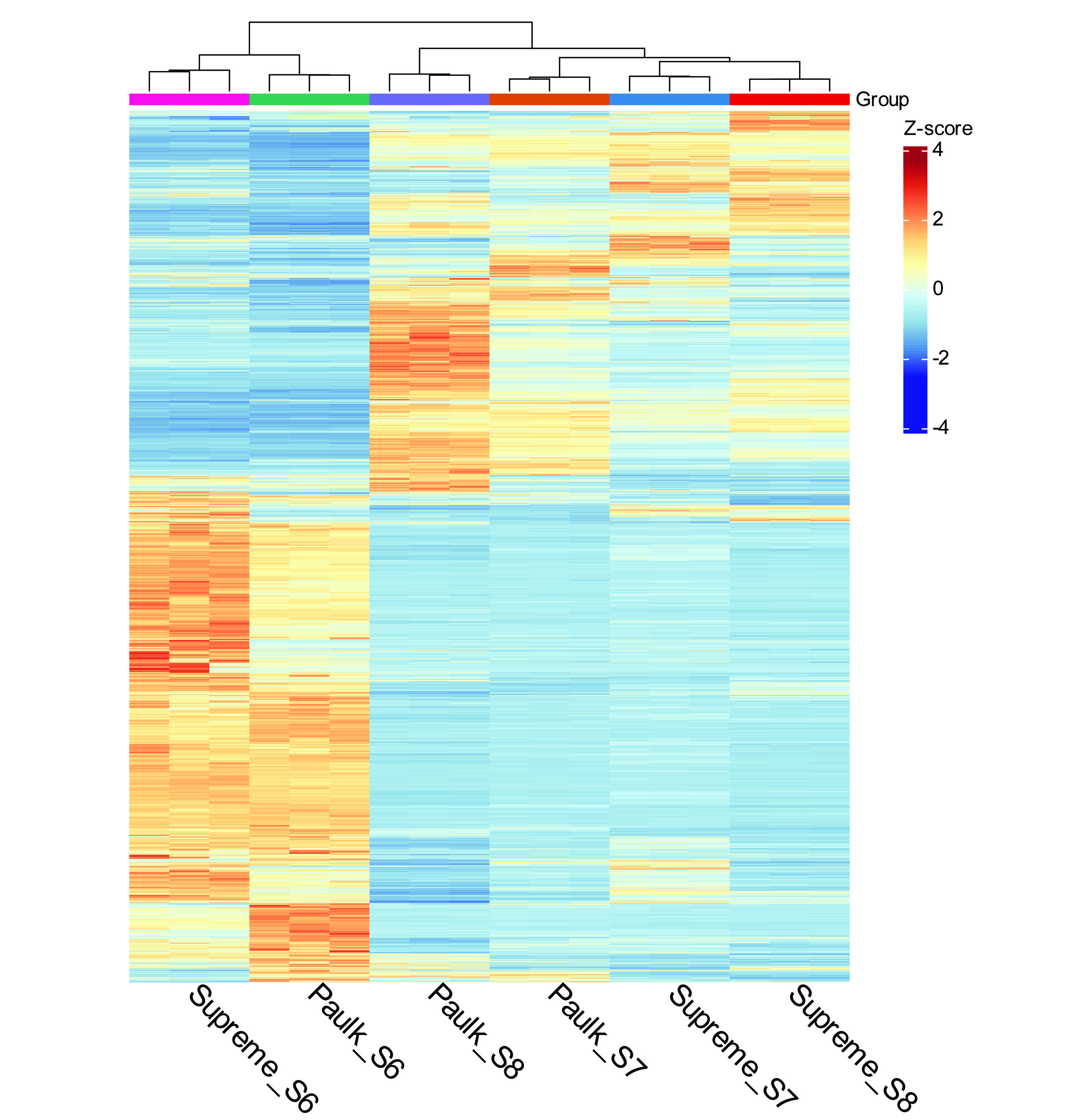

Supplement: Supplementary file 1 [file plants-14-02025-s001.zip › supplementary data/Fig S2 HCA transcriptome.jpg]

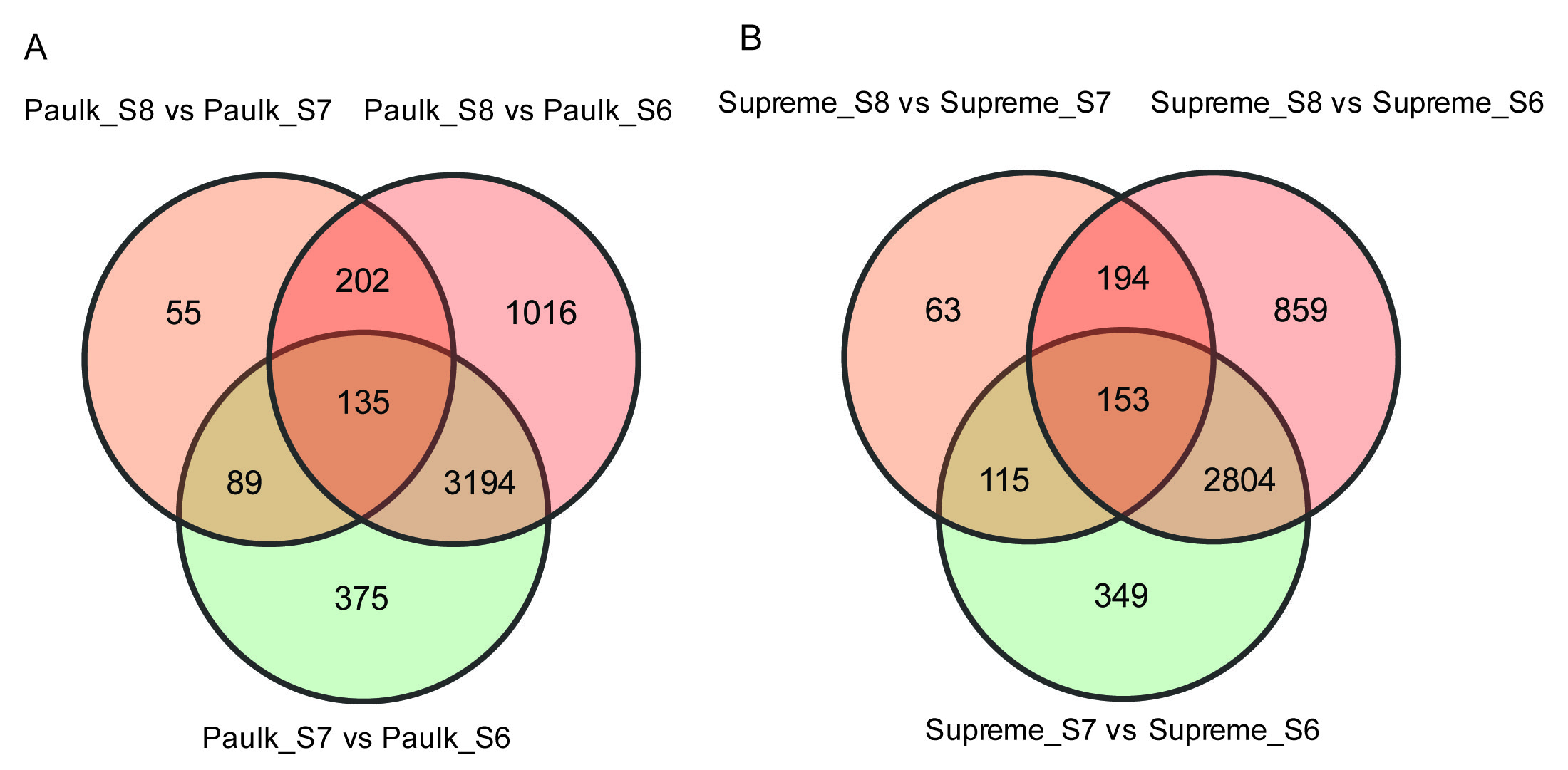

Supplement: Supplementary file 1 [file plants-14-02025-s001.zip › supplementary data/Fig S3 Veen geness-01.jpg]

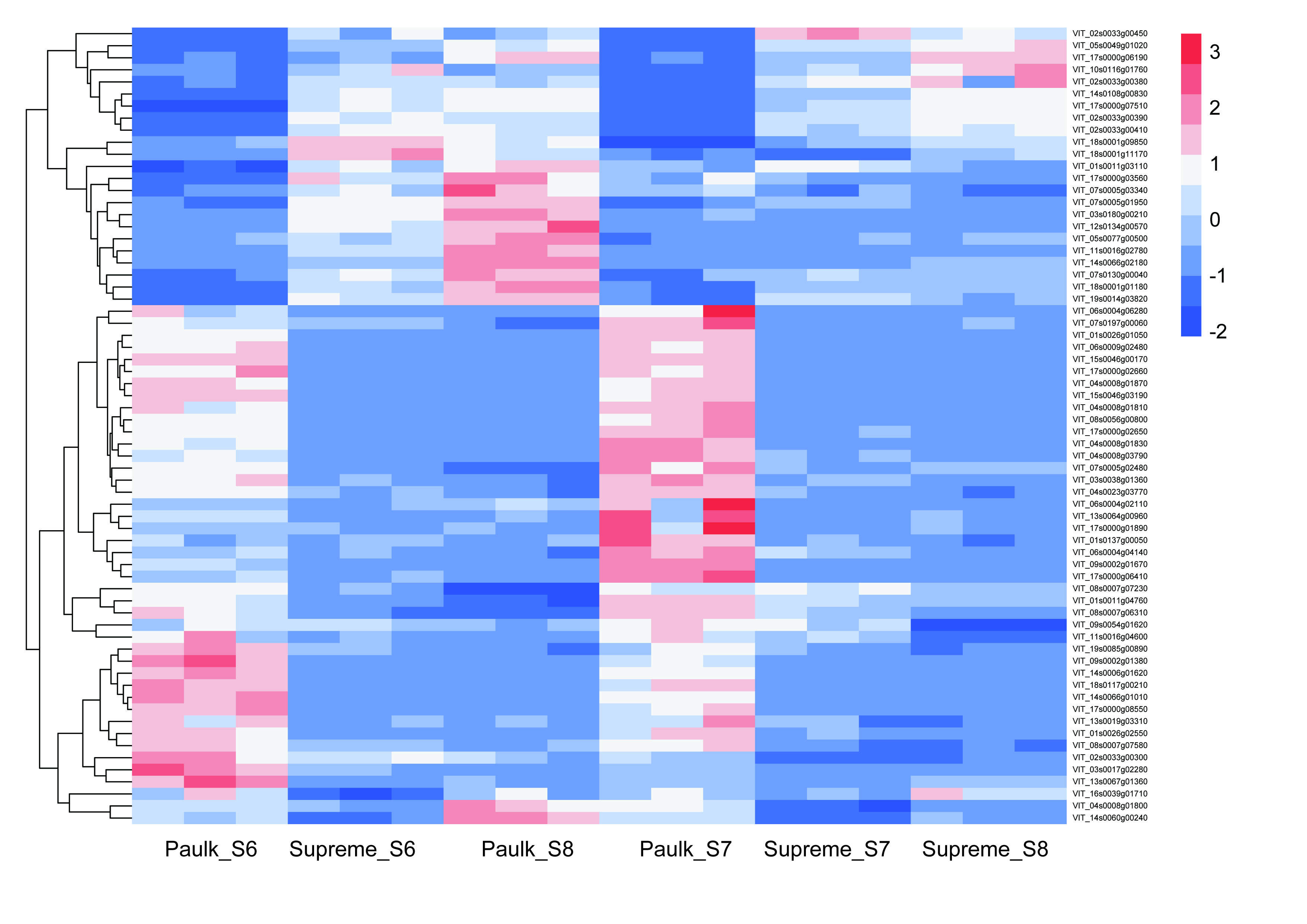

Supplement: Supplementary file 1 [file plants-14-02025-s001.zip › supplementary data/Fig S4 MYB clustering analysis.jpg]

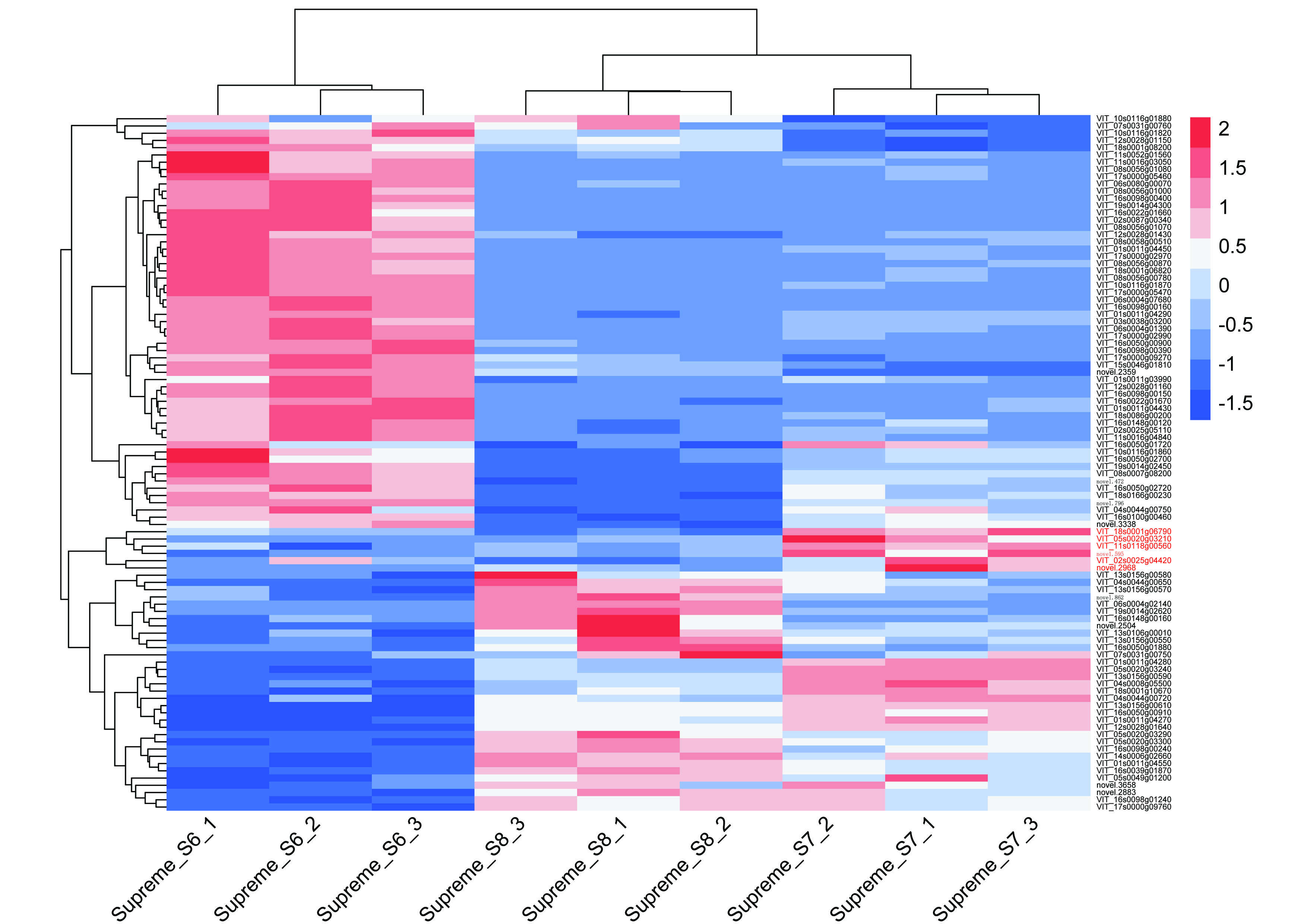

Supplement: Supplementary file 1 [file plants-14-02025-s001.zip › supplementary data/Fig S5 MATE genes clustering.jpg]
